# The influence of fibrillin‐1 and physical activity upon tendon tissue morphology and mechanical properties in mice

**DOI:** 10.14814/phy2.14267

**Published:** 2019-11-13

**Authors:** Peter H. T. Tran, Tanja Skrba, Elisabeth Wondimu, Giuseppina Galatioto, René Brüggebusch Svensson, Annesofie T. Olesen, Abigail L. Mackey, S. Peter Magnusson, Francesco Ramirez, Michael Kjaer

**Affiliations:** ^1^ Department of Pharmacological Sciences Icahn School of Medicine at Mount Sinai New York New York; ^2^ Institute of Sports Medicine Department of Orthopedic Surgery M Bispebjerg Hospital Copenhagen Denmark; ^3^ Center for Healthy Aging Faculty of Health and Medical Sciences University of Copenhagen Copenhagen Denmark; ^4^ Department of Physical and Occupational Therapy Bispebjerg Hospital Copenhagen Denmark; ^5^ Department of Biomedical Sciences Faculty of Health and Medical Sciences University of Copenhagen Copenhagen Denmark

**Keywords:** Tendinopathy, Exercise, Marfan, Survival, Biomechanics

## Abstract

Fibrillin‐1 mutations cause pathological changes in connective tissue that constitute the complex phenotype of Marfan syndrome. In this study, we used fibrillin‐1 hypomorphic and haploinsufficient mice (Fbn1^mgr/mgR^ and Fbn1^+/−^ mice, respectively) to investigate the impact of fibrillin‐1 deficiency alone or in combination with regular physical activity on tendon tissue morphology and mechanical properties. Morphological and biomechanical analyses revealed that Fbn1^mgr/mgR^ but not Fbn1^+/−^ mice displayed smaller tendons with physical properties that were unremarkable when normalized to tendon size. Fbn1^mgR/mgR^ mice (*n* = 43) Fbn1^+/−^mice (*n* = 27) and wild‐type mice (WT, *n* = 25) were randomly assigned to either control cage conditions (*n* = 54) or to a running on a running wheel for 4 weeks (*n* = 41). Both fibrillin‐1‐deficient mice ran voluntarily on the running wheel in a manner similar to WT mice (3–4 km/24 h). Regular exercise did not mitigate aneurysm progression in Fbn1^mgR/mgR^ mice (*P* < 0.05) as evidenced by unmodified median survival. In spite of the smaller size, tendons of fibrillin‐1‐deficient mice subjected to regular exercise showed no evidence of overt histopathological changes or tissue overload. We therefore concluded that lack of optimal fibrillin‐1 synthesis leads to a down regulation of integrated tendon formation, rather than to a loss of tendon quality, which also implies that fibrillin‐1 deficiency in combination with exercise is not a suitable animal model for studying the development of tendon overuse (tendinopathy).

## Graphical abstract

Regular exercise did not worsen or improve the median survival of Marfan syndrome mice, and they showed no signs of pathological changes in their tendons. Marfan syndrome mice had smaller tendons than wild type while the mechanical properties remained unchanged. Taken together, substantial fibrillin‐1 deficiency in Marfan syndrome does affect tendon quantity rather than quality regardless of regular exercise.

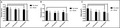

## Introduction

Mutations in the extracellular matrix (ECM) protein fibrillin‐1 cause pathological changes in the connective tissue and results in the pleiotropic manifestations of Marfan syndrome (MFS), which includes thoracic aortic aneurysm (TAA) (Ramirez et al., 2018). It is unknown to what extent tendon tissue is influenced by fibrillin‐1 deficiency. While only 2% of tendon dry weight is composed of elastic fibers compared to 60–80% collagen fibers, fibrillin‐1 might still play an important role for tendon adaptation to mechanical loading (Thorpe et al., 2012). Despite the impressive tensile strength of tendons, overloading of tendon tissue in humans in relation to occupational‐ or leisure activity represents a significant clinical and socioeconomic burden, but our knowledge regarding the pathogenesis of tendinopathy limited (Magnusson et al., 2010; Heinemeier et al., 2013; Pingel et al., 2014; Dakin et al., 2015). For example, we still do not know whether voluntarily running in MFS results in a beneficial physiological adaptation of tendon tissue or possibly in a tendinopathic phenotype due to tissue overloading. It is equally unclear what the impact of voluntarily running might be on cardiovascular disease in MFS. Contrary to the believe that increased blood pressure during voluntarily running would exacerbate TAA progression, it has recently been reported that moderate exercise actually decreased the rate of aneurysm growth in mice with a mild non‐lethal form of MFS (Gibson et al., 2017; Thijssen et al., 2019). Unfortunately there are no data in the literature of controlled trials that have longitudinally evaluated how exercise influences TAA progression in MFS patients (Thijssen et al., 2019).

To address the aforementioned issues, we analyzed biomechanical and histological properties of tendons isolated from wild‐type (WT) mice and mice with different degrees of fibrillin‐1 deficiency (50 and 80% deficiency) that had been subjected to either control cage conditions or voluntary running. The results of our experiments demonstrated that fibrillin‐1‐deficient mice tolerated voluntarily running comparable to WT mice without a change in median survival or any sign of tendon histopathology or tissue overload.

## Methods

### Animals

All experiments used 3‐ to 4‐week‐old male fibrillin‐1‐deficient mice and sex‐and age‐matched WT littermates maintained on the C57BL/6J genetic background. Fibrillin‐1‐deficient mice included Fbn1^mgR/mgR^ mice, which produce ~ 20% of normal fibrillin‐1 and display early onset progressively severe MFS (median survival 3 months), and Fbn1^+/−^ mice, which produce ~ 50% of normal fibrillin‐1 and show no appreciable clinical signs of MFS and have a normal life span (Pereira et al., 1997; Carta et al., 2009). The Institutional Animal Care and Use Committees of the Icahn School of Medicine at Mount Sinai in New York City reviewed and approved all animal studies.

### Experimental setup

The mice were randomized to either control (cage with no access to running wheel) or to wheel‐running regimen (cage equipped with running wheel). The period of voluntarily running or sedentary control was from week 4 to week 8 when the mice were euthanatized for tissue collection. Mice in the wheel‐running group were single‐housed with a custom‐made running wheel and had voluntary access to the running wheel during both diurnal and nocturnal cycles. The running wheel consisted of a 0.11‐m diameter wheel connected to sampling box and could rotate freely in the cage. Mice were housed in polycarbonate regular temperature cages (dimensions 257 mm × 483 mm × 152 mm) (PC10196‐RT, Allentown) with wire bar lids in stainless steel (WBL 1019 MMB, Rim Rod Design, Allentown). The lid was lifted 10 mm to fit the running wheel and have it run freely. There was an internal resistance in the running wheel system of 1.45 g on average (min: 1.03 g and max: 2.00 g) and therefore all running wheels were calibrated to have a resistance of 2.00 g using lead weights in the sampling box. Wheel revolutions would rotate a plastic plate with four holes inside the sampling box, allowing diode light to pass through the holes and into a light receiver. Thus, it was possible to count every 45° degrees rotation as a light impulse (light–no light), eight impulses would equal one revolution. Data samples were automatically stored on local SD cards at 1 Hz and collected by the investigators at 9 am every day when the mice were least active. The lights in the animal facility were turned off at 7 pm and back on at 7 am to mimic a nocturnal lifestyle. All cages had bedding and *ad libitum* access to food and water. The temperature in the facility was fixed at 22°C. Due to a power outage seven mice had insufficient data for analysis and were omitted from the results. The remaining data were analyzed in Matlab (R2017a, Mathworks) to obtain distance and velocity. Average velocity was calculated as the number of revolutions if the wheel rotated more than a set threshold of 45° during 1 sec and divided by time passed until the wheel was hitting the threshold. The number of revolutions was converted to distance using the formula for circumference of a circle. All procedures and animal care were approved by The American Association for Laboratory Animal Science (IACUC).

### Tissue sampling

After euthanizing at 8 weeks with carbon dioxide (100% at flow 3 L/min) the Achilles and flexor digitorum longus tendons of the forelimbs were dissected free of other tissues and the hindlimbs were collected with all tissue intact for histology. The flexor digitorum longus tendon was packed in gauze with PBS (phosphate‐buffered saline) and stored at −80°C before biomechanical testing. The Achilles tendons for histology were formalin‐fixed as whole limbs in 4% paraformaldehyde for 48 h at 4°C and were placed in a vertical position with the ankle in neutral position and the femur in 45° to tibia (Cikach et al., 2018). Sections of 2 micrometer were cut using a microtome and then placed in a 37° Celsius water bath to be picked up by superfrost plus glass slides. An ice bucket with dry‐ice was placed on top of the microtome to cool down the paraffin block during sectioning and cold forceps were used to handle the sections. Slides were left to dry overnight at 32°C. Each limb provided approximately two to four sections per slide and 30–35 slides in total. An estimated 50% of the sections were not usable for histochemistry either because they were shredded by the microtome because of calcified bone present in the sample or due to suboptimal transfer from microtome to the water bath. Necropsy was a standardized procedure for all mice and confirmed the diagnosis of aortic aneurysms in Fbn1^mgR/mgR^ mice that was lost during the experiment.

### Biomechanical testing

Each end of the flexor digitorum longus tendon was dried and glued to clamps, using commercial superglue. The middle part of the tendon was kept moist by wrapping it in a thin piece of gauze soaked in PBS. The clamps were part of a custom‐made Deben mechanical rig (20N tensile stage, Petri dish version, Deben Ltd, Stuffolk, UK). Deben software was used to control movement of the clamps. The setup was then secured to actuators of the linear mechanical testing machine with the tendon sub‐emerged in PBS. To remove any initial slack of the tendon before testing 0.1N was applied to the tendon and the length of the tendon was measured as distance from clamp to clamp. Testing protocol consisted of 6 preconditioning cycles, 10 testing cycles and then finished with a maximum test, pulling the clamps apart until the tendon broke. Precondition‐phase was necessary since the tendons had been thawed from 80°C and this process could alter the fiber alignment. Thus, tendons would become slack again after precondition and had to be recalibrated with 0.1N. After recalibration the tendon length and width were measured. Tendons were tested at 2.5% strain and 4 mm/second (determined by pilot study) through all cycles. Sampling time was set to 10 Hz through the duration of tests. Specimens were visualized and recorded using an Olympus light microscopy with Leica camera attached. Force‐ and position data were obtained from the Deben Software during mechanical testing. Values were normalized with tendon dimensions to stress–strain graphs using excel. Mechanical properties, such as max force and stiffness, that are dependent on tissue dimensions and material properties, such as modulus and hysteresis, that are independent of tissue dimensions were analyzed.

### Collagen alignment

Sections were stained with Picrosirius red (Direct red 80, CAT#365548, Sigma‐Aldrich) to visualize collagen fibers using a standard protocol (Kiernan,^2014^; Puchtler et al., 1973; Junqueira et al., 1979). Briefly, sections were deparaffinated with xylene before hydration in decreasing ethanol solutions. Afterward, sections were stained in Picrosirius red working solution (1 mg Sirius red added to 1 L 1.3% (w/v) picric acid solution in diH_2_O) at room temperature for 1 h and then washed two times in glacial acetic acid. Sections were rehydrated with ethanol before being mounted with Cytoseal® and coverslips. A brightfield microscope equipped for polarization microscopy (Axio Lab.A1, Zeiss, Germany) was used for visualization under 20x zoom (20x/0.45 Pol, N‐Achroplan, 0.227 7 µm/pi, Zeiss, Germany): Red for mature collagen fibers and cyan for unmatured collagen fibers. The stage was manually rotated in incremental degrees of 10° from 0° to 180° to observe changes in color intensity and pictures were then taken for every 10° using a mounted 6Mpx camera (AxioCam 506, Zeiss, Germany). Pictures were taken with the software ZEN Lite (Zen Lite 2.3, Windows, Zeiss) with the following configurations: Camera acquisition ROI size 2048 and offset 35,280. Exposure set to 20,000 ms with 10 cycles (time series). Data analysis of the pictures was done in Fiji ImageJ2 (2.0.0‐rc‐69/1.52i, macOs) by measuring the color intensity of red and green. Fibers that were aligned under polarized light would display an intensive red light while fibers arranged 90° to the light would become dim. Rotating from 0° to 360° would vary the light intensity of the sample and therefore was plotted as a sinus‐curve with 2π period. Thus, maximum light intensity is given a value of 1 or −1 on the y‐axis while 0 is for minimum light intensity (dim). Given that every periodic π (180°) would give the same signal as 0°, π/2 would reveal maximum light intensity and the percentage of light intensity was measured as relative to 2π. This takes into account that absolute lack of light or dimness is not achievable because some fibers will always be aligned to a given angle. An absolute 100% light intensity is an expression of completely unreflective light passing through the sample and is neither possible due to some fibers being out of alignment.

### Statistics

Mantel–Cox test (logrank test) was used to analyze the survival rate of mice in wheel‐running and control group within genotypes. A one‐way ANOVA was used to analyze running‐data for effect of genotype on distance and velocity. For other data a two‐way ANOVA was performed to determine any effects of genotype, groups, and their interaction on tendon composition, dimensions, and mechanical properties. If the two‐way ANOVA was significant, individual differences within genotype and groups were tested with a post hoc test (Tukey). Statistical analysis was performed in Prism (GraphPad, Prism 8.0.1, MacOs) and in all cases, a p‐value less than 0.05 was considered significant. Data are presented as mean ± standard error of the mean unless indicated otherwise in text under figure or table.

## Results

### Voluntarily aerobic running and survival

**Figure 1:**
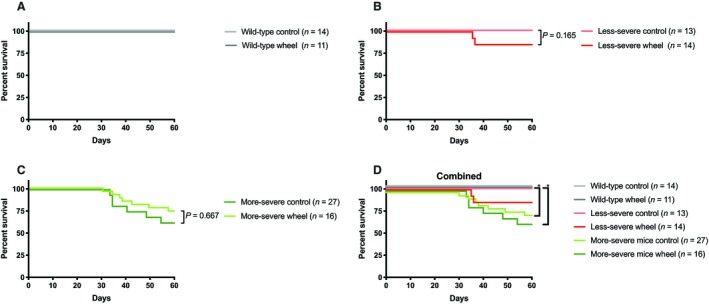
30‐day survival‐curves for all three genotypes. (A) wild‐type mice. (B) Less‐severe MFS mice. (C) More‐severe MFS mice.( D) All mice genotypes. There was no difference in survival between wheel running‐group and controls in any of the groups. More‐severe MFS mice had a median survival of 10 weeks regardless of wheel running and therefore showed significantly lower survival‐rate when compared to wild‐type. ^*^ *P*<0.05.

There was no significant difference between genotypes for running distance and average running velocity (Table 1). Most of the running occurred between 10 PM and 9 AM, approximately three hours after the lights of the animal facility were switched off (data not shown). All groups with access to running wheel ran 3–4 km/24 h (Table 1). There was no significant change in the survival rate of Fbn1^mgR/mgR^ mice relative to the same mutant animals under resting conditions (Fig. 1).

**Table 1:** Running data over 30 days for mice on running‐wheel.

Experimental groups	Number of mice	Distance (km) Mean ± SE	Average velocity (m/sec) Mean ± SE
Wild‐type	11	116 ± 22	0.20 ± 0.02
Less‐severe MFS	14	102 ± 15	0.21 ± 0.01
More‐severe MFS	14	89 ± 13	0.22 ± 0.01

Distance (km) was calculated as total distance during the 30‐day period. Average velocity (m/s) is a measured distance for each running‐bout that resulted in a rotation of the wheel more than 45° during 1 sec divided by time until it no longer exceeded the threshold of 45° per second. SE, standard error of mean.

### Tendon morphology and mechanical properties

**Figure 2:**
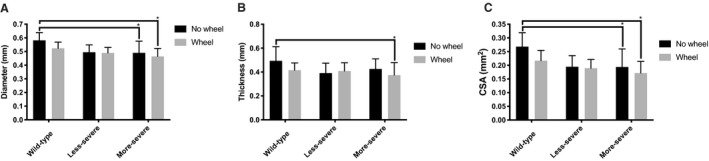
Tendon dimensions of mice in both wheel‐running and control group. (A) Diameter (mm). (B) Thickness (mm). (C) Cross‐sectional area (CSA, mm^2^). Diameter, thickness, and cross‐sectional are of more‐severe MFS mice were significant less than that in wild‐type. There was no significant difference of less‐severe MFS mice when compared to other genotypes regardless of groups. ^*^ *P*<0.05. In control group: N_Wild‐type_ = 14, N_Less‐severe_ = 12, and N_More‐severe_ = 9. In wheel‐running‐group: N_Wild‐type_ = 11, N_Less‐severe_ = 11, and N_More‐severe_ = 10.

**Figure 3:**
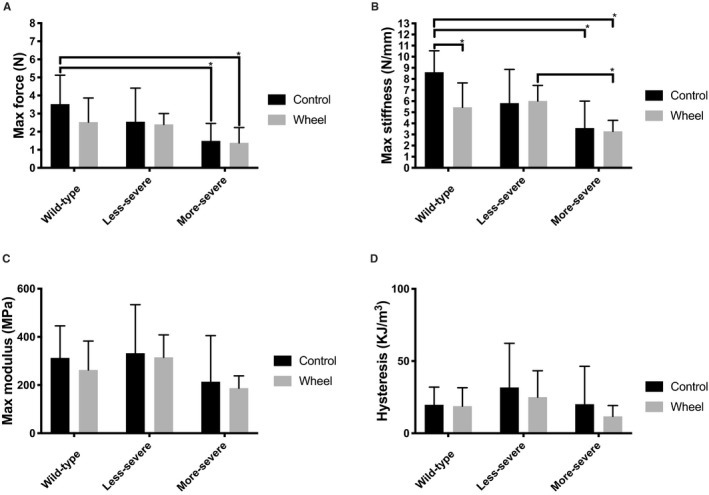
Mechanical data from genotypes of control and wheel‐running group. (A) Maximal force (N). (B) Maximal stiffness (N/mm). (C) Maximal modulus (MPa). (D) Hysteresis (KJ/m^3^). More‐severe mice had significantly lower max force and max stiffness when compared to wild‐type. ^*^ *P*<0.05. In control group: N_Wild‐type_ = 13, N_Less‐severe_ = 12, and N_More‐severe_ = 9. In wheel‐running group: N_Wild‐type_ = 11, N_Less‐severe_ = 11, and N_More‐severe_ = 11.

**Figure 4:**
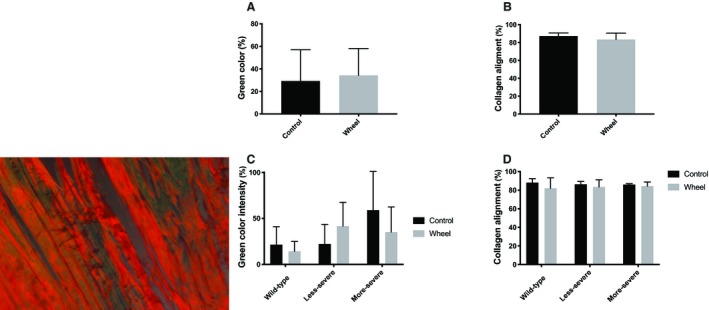
Results from Picrosirius staining of genotypes in control and wheel running‐group. ( Left panel image: example image). (A) Overall green color (%). (B) Overall collagen alignment (%). (C) Green color intensity (%). (D) Collagen alignment (%). Left panel image: example image. In control group: N_Wild‐type_ = 8, N_Less‐severe_ = 4, and N_More‐severe_ = 3. In wheel‐running group: N_Wild‐type_ = 3, N_Less‐severe_ = 7, and N_More‐severe_ = 5.

There was a significant difference in baseline tendon dimensions between genotypes (Fig. 2). Fbn1^mgR/mgR^ tendons exhibited a smaller cross‐sectional area (CSA) due to a smaller width and a decreased anterior–posterior thickness compared to the tendons of WT mice (*P* < 0.05) (Fig. 2A–C). By contrast, the dimensions of Fbn1^+/−^ tendons were comparable to those of WT mice (*P*> 0.05). In all three groups of mice, voluntarily running had appreciable impact on tendon morphology (Fig. 2). Fbn1^mgR/mgR^ mice had the weakest tendons of all genotypes regardless of voluntarily running (Fig. 3). Tendon max force was statistically lower in Fbn1^mgR/mgR^ compared to WT mice (*P* < 0.05) but was similar between Fbn1^mgR/mgR^ ‐ and Fbn1^+/−^ mice (Fig. 3A). By contrast, max force in less severe mice of both resting and wheel‐running groups was not different than WT mice (Fig. 3A). The maximal stiffness was significantly lower in Fbn1^mgR/mgR^ mice than WT mice (Fig. 3B) regardless of level of physical activity level. Finally, Fbn1^+/−^ mice had lower max stiffness than WT mice but higher max stiffness than Fbn1^mgR/mgR^ mice in the wheel running‐group (*P* < 0.05)(Fig. 3B). An effect of voluntarily running upon tendon max stiffness was only observed in the WT group, as evidenced by decreased tendon stiffness (*P* < 0.05) (Fig. 3B). When stiffness was normalized to tendon dimensions max modulus values were similar between genotypes and wheel‐running groups (Fig. 3C). In addition, hysteresis remained similar between genotypes and was not modified by voluntarily running (Fig. 3D). Finally, there was no significant difference between genotypes and groups in other parameters of tendon mechanics (data not shown). Immature collagen and fiber alignment analyzed with immunohistochemistry were not significantly different between any of the genotypes (Fig. 4). Also, there was no significant effect of voluntarily running on the quality of tendons in any of the groups, and even when genotypes were pooled together in either a sedentary control or in a running wheel‐group no difference was observed between active and sedentary mice (Fig. 4A,B).

## Discussion

Three are the major new findings of our study. First, substantial fibrillin1 deficiency affected the size rather than the quality of Fbn1^mgR/mgR^ tendons. Second, smaller tendons did not display pathological changes when loaded with voluntary running. Third, voluntarily aerobic running did not improve (or worsen) the median survival of Fbn1^mgR/mgR^ mice.

Elastic fibers are made of a central core of elastin surrounded by fibrillin‐rich microfibrils (Heinemeier et al., 2013). Unlike in the aorta, elastic fibers are a minor structural component of the tendon matrix. In spite of being part of the same ECM macroaggregate, elastin and fibrillin‐1 deficiency have very different effects of tendon physiology. In this study, Fbn1^mgR/mgR^ mice displayed markedly smaller tendons than WT mice due to reduced anterior–posterior thickness and width diameter (Fig. 2), which was associated with unaltered modulus and collagen orientation. The haploinsufficient elastin mice (Eln^+/−^mice) on the other hand, showed no significant reduction in Achilles tendon size, but had an increased mechanical stiffness (Eekhoff et al., 2017). Interestingly, in the present study tendons from Fbn1^mgR/mgR^ were less stiff than their healthy counterparts. Tendons from Fbn1^mgR/mgR^ were expected to become stiff as there is more complete engagement of collagen fibers to tendon loading, which has been shown in other studies on elastin haploinsufficiencient mice (Li et al., 1998; Carta et al., 2009; Eekhoff et al., 2017). It has been shown that there is a significant increase in the linear stiffness in Eln^+/−^ in the absence of a change in other mechanical parameters (Eekhoff et al., 2017). In the present study, it appeared that tendons from more‐severe fibrillin‐deficient (Fbn1^mgR/mgR^) mice seem longer than wild type (data not shown). This suggests a role of elastin in collagen crimp with tendons becoming slacker if elastin is somehow compromised (Henninger et al., 2015). A potential shifting of the stress–strain curve from right to left would minimize the physiological normal range of such tendons, thus also explains why maximum force was lowest in Fbn1^mgR/mgR^. However, hysteresis was unaffected by genotype and exercise, indicating that a secondary source of elastic recoil might be present (Henninger et al., 2013). It seems that deficiency of elastin and fibrillin, respectively, does influence mechanical properties somewhat different and interact with each other when it comes to mechanical properties of the tendon (Henninger et al., 2015). Taken at face value, these findings suggest that elastin primarily influences the mechanical properties rather than the size of tendons, whereas fibrillin‐1 is predominantly involved in optimizing tendon growth rather than influencing tissue mechanics (Fig. 3) (Ramirez and Pereira, 1999). The different responses of elastin or fibrillin‐1‐deficient tendons to voluntarily running induced loading is another example of the distinct roles that these elastic fiber components play a role in tendon physiology.

Pathological changes in tendon tissue were not observed after voluntary running in either mice producing half of the normal amount of fibrillin‐1 (Fbn1^+/−^ mice) or those making only 20% of it (Fbn1^mgR/mgR^ mice). By contrast, degradation of elastic fibers has been associated with clinical signs of chronic tendinopathy (overuse‐injuries) in Eln^+/−^ mice (Wu et al., 2016; Wu et al., 2017). While the underlying mechanism remains unknown, differentiation of functions by the two major components of the elastic fibers has also been reported in the mouse arteries (Carta et al., 2009). The lack of pathological changes in tendon of fibrillin deficient mice indicates that the use of this mouse model in combination with voluntary running is not optimal for studying tendon overuse injury. While we cannot rule out that a higher mechanical work load than used in the present study could have caused pathological tendon changes, the fact that fibrillin‐1‐deficient mice had lower tendon diameter (but similar body weight), but ran a similar distance as healthy mice exclude that these mutant animals could be used as an experimental model to study tendinopathy pathogenesis. In humans, tissue remodeling in tendons is limited, and more chronic physiological, or pathological adaptation of tendon required several months (Heinemeier et al., 2013). In the present study on mice, we chose the 60‐day period to ensure that a significant number in the diseased group would still be alive.

Our last new finding concerns the relationship between cardiovascular function and wheel running in MFS. It was recently reported that mild aerobic wheel running (2–5 km/day) alleviated the degree of vessel degeneration in mice with a non‐lethal form of MFS in which the ascending aorta dilates but does not rupture (Fbn1^C1039G/+^ mice)(Gibson et al., 2017). However, we found no modifications in aneurysm progression to dissection and premature death when our mice with lethal MFS (Fbn1^mgR/mgR^ mice) were subjected to a similar running regimen (3–4 km/day). Interestingly, a running intensity up to 65% *V*_O2max_ showed protective effects against elastin fiber fragmentation within the aortic wall which supports the notion that other factors than volume may determine optimum exercise regime. We have no clear explanation for the discrepancy between the two studies short of noting that median survival (and implicitly aneurysm dissection and rupture) is a more robust read out of running‐induced modifications of arterial disease than elastic fiber fragmentation.

## Limitations

Most studies on MFS have focused on blood vessels in animal knockout models and rarely on changes in tendon (Pereira et al., 1997; Li et al., 1998; Pereira et al., 1999; Galatioto et al., 2018; Ramirez et al., 2018). However, blood vessels and tendons are distinct structures with the former containing much more elastic fibers (Kannus, 2000; Shinaoka et al., 2013; Pang et al., 2017), and therefore a direct comparison to previous studies is difficult. Fibrillin‐1 may play a role in elastogenesis, which has been shown in blood vessels (Carta et al., 2009) but never in loaded tendons according to our knowledge. Unfortunately, elastin content was not measured in tendons, which would have provided valuable information.

In the current study, the exercise period was 30 days, and it should be noted that the severe animal‐model have an inferior survival rate due to aortic complications (Pereira et al., 1997; Pereira et al., 1999) with a median survival rate of only 90 days (Pereira et al., 1999). It is therefore possible that 30 days of voluntarily exercise was insufficient to see any pathological change in tendons.

In conclusion, our findings correlated fibrillin‐1 deficiency with the development of smaller but morphologically and biomechanically normal tendons that can tolerate voluntarily running loading to the same extent as the WT counterparts. Furthermore, we found that mice with severe MFS tolerated voluntarily aerobic running without developing tendinopathy or exacerbating aneurysm progression.

## Conflict of Interest

None declared.
